# Natural curcuminoids encapsulated in layered double hydroxides: a novel antimicrobial nanohybrid

**DOI:** 10.1186/s13065-016-0179-7

**Published:** 2016-05-31

**Authors:** Ajona Megalathan, Sajeewani Kumarage, Ayomi Dilhari, Manjula M. Weerasekera, Siromi Samarasinghe, Nilwala Kottegoda

**Affiliations:** Institute of Chemistry, College of Chemical Sciences, Welikada, Rajagiriya, Sri Lanka; Department of Chemistry, Faculty of Applied Sciences, University of Sri Jayewardenepura, Gangodawila, Nugegoda, Sri Lanka; Department of Microbiology, Faculty of Medical Sciences, University of Sri Jayewardenepura, Gangodawila, Nugegoda, Sri Lanka; Center for Excellence in Nanotechnology, Nanoscience and Technology Park, Sri Lanka Institute of Nanotechnology, Pitipana, Homagama, Sri Lanka; Advanced Materials Research Center, Faculty of Applied Sciences, University of Sri Jayewardenepura, Gangodawila, Nugegoda, Sri Lanka

**Keywords:** Layered double hydroxide, Curcuminoids, Curcumin, Turmeric, Antimicrobial, Slow release, Nanohybrid

## Abstract

Currently, there is an increased scientific interest to discover plant based drug formulations with improved therapeutic potential. Among the cornucopia of traditional medicinal plants, *Curcuma longa* rhizomes have been used as a powerful antibacterial and antifungal agent. However, its practical applications are limited due to its instability under thermal and UV radiation and its low bioavailability and the extensive procedures needed for isolation. This study focuses on exploring the potential of nanotechnology-based approaches to stabilize the natural curcuminoids, the major active components in turmeric without the need for its isolation, and to evaluate the release characteristics, stability and antimicrobial activity of the resulting nanohybrids. Natural curcuminoids were selectively encapsulated into nanolayers present in Mg–Al-layered double hydroxides (LDHs) using a method that avoids any isolation of the curcuminoids. The products were characterized using solid state techniques, while thermal and photo-stability were studied using thermogravimetric analysis (TGA) and UV exposure data. The morphological features were studied using scanning electron microscope (SEM) and transmission electron microscope (TEM). Drug release characteristics of the nanohybrid were quantitatively monitored under pH 3 and 5, and therapeutic potentials were assessed by using distinctive kinetic models. Finally, the antimicrobial activity of curcuminoids-LDH was tested against three bacterial and two fungal species. Powder X-ray diffraction, Fourier transform infra-red spectroscopy, SEM and TEM data confirmed the successful and selective encapsulation of curcuminoids in the LDH, while the TGA and UV exposure data suggested the stabilization of curcuminoids within the LDH matrix. The LDH demonstrated a slow and a sustained release of the curcuminoids in an acidic medium, while it was active against the three bacteria and two fungal species used in this study, suggesting its potential applications in pharmaceutical industry.

**Electronic supplementary material:**

The online version of this article (doi:10.1186/s13065-016-0179-7) contains supplementary material, which is available to authorized users.

**Graphical abstract:**
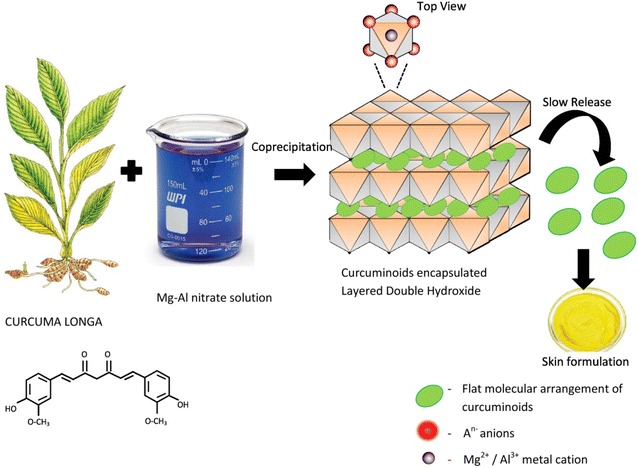
Synthesis of Curcuminoid-LDH by coprecipitation method and the slow release process of curcuminoids from LDH matrix

## Background

The discovery of therapeutic potential of plant derived remedies based on traditional medicine has raised renewed interest in the development of drugs from natural sources. In this context, many attempts have been focused on integrating traditional medicine into western drug formulations. Despite the known challenges associated with the development of a potent drug from natural biomolecules the recent revival of interest in these molecules has resulted in broad interdisciplinary research approaches to plant based drug discovery. Among the cornucopia of traditional medicinal plants, *Curcuma longa* rhizomes are known to have various therapeutic properties, including antibacterial and antifungal activity. Curcumin, 1, 7-bis (4-hydroxy-3-methoxy-phenyl)-1, 6-heptadiene-3, 5-dione, the main coloring substances in turmeric, and two related compounds, demethoxycurcumin (DMC) and bisdemethoxycurcumin (BDMC), are collectively known as curcuminoids, which have well-known antimicrobial properties together with highly potent, non-toxic, bioactive characteristics. Among the many common health related issues, infectious diseases and emerging microbial species with resistance to common antimicrobial agents represent a significant burden to the healthcare systems [1]. In treatment of such diseases, curcuminoids would be a potential candidate. However, these curcuminoids suffer from low aqueous solubility, poor bioavailability, and low stability and therefore, have limited practical use, necessitating the modification of their properties in order to develop a versatile, useful and effective therapeutic product [2].

In this realm, nanotechnology has shown much promise in pharmaceutical industry [3]. Among the many available nanomaterials, an immense deal of attention has been focused on nanolayered inorganic materials because of their ability to encapsulate and immobilize various organic and inorganic molecules as well as bio molecules in the interlayer space due to their fascinating lamellar structures [4]. In addition, the structural and morphological tunability, convenient synthesis, versatility and their low toxicity, with good biocompatibility and bio-degradability have resulted in high intrinsic pharmacological activity compared with conventional drugs and other controlled- and slow-release drugs [5–9]. The positive surface charge of an LDH layer is due to the partial substitution of divalent cations (Mg, Zn, Ni, etc.) for trivalent cations (Al, Cr, etc.), thus making it viable for the intercalation of negatively charged drugs or biomolecules such as DNA [10, 11]. A controllable sustained anion exchange that is pH dependent is possible due to the structure of LDHs, which is also mandatory for the controlled-release properties of this system, making it a valuable candidate for biological and pharmaceutical applications [12].

Most of the work on LDH-based drug delivery systems has been based on already existing active drugs, while little attention has been devoted to exploring the potential of the encapsulation of naturally occurring biologically active compounds in slow- and controlled-release applications. Samindra and Kottegoda [13] have reported the successful intercalation of chemically isolated curcumin (CIC) into LDH and demonstrated its slow release behavior [14]. A recent attempt has been made to evaluate the anticancer activity of curcumin—LDH [15]. Perera et al. [16] have also reported the potential of citrate intercalated LDH as an antimicrobial active formulation in body lotions and have verified its activity against *Candidaspecies*. In recent literature Shafiei et al. [17] have reported the successful encapsulation of epigallocatechin gallate into layered double hydroxide and it’s in vitro anti-tumor properties.

However, none of the previous work has reported the selective encapsulation of natural pharmaceutically active compounds into layered double hydroxides. This study is an extension to the work done by Samindra and Kottegoda [13] on chemically isolated curcumin encapsulated LDHs. This study lays the foundation for the successful and selective encapsulation of curcuminoids into nanolayers present in LDHs without the need for isolation unlike previous attempts which involved isolation from crude turmeric. In addition, the encapsulation process is expected to improve its photo stability, water solubility, and prolonged bio-availability thus allowing it to be used in broad spectrum of medical applications.

## Results and discussion

### Identification of curcuminoids

**Fig. 1:**
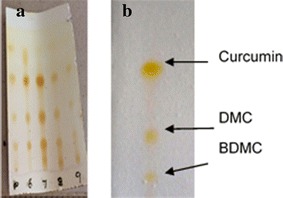
TLC of **a** turmeric powder and **b** curcuminoids-LDH (SEC-LDH). TLC was conducted by dissolving turmeric/curcuminoid-LDH (selectively encapsulated) in acetone and the mobile phase was a mixture of chloroform (95 %) and methanol (5 %)

According to the thin layer chromatography (TLC) of turmeric powder dissolved in acetone, several spots were observed, thus signifying the presence of other components, such as protein, carbohydrates, fat, minerals, other than curcuminoids. The TLC of the de-intercalation of curcuminoids from LDH shows only three spots, which represent the presence of curcumin, DMC and BDMC with RF values of 0.75, 0.55, and 0.27, respectively. These RF values compare well with those reported for curcumin, DMC and BDMC in a previous work [18]. Furthermore, the highest intense peak corresponds to curcumin, which is the major component in natural turmeric. These observations suggest that curcuminoids have selectively intercalated into the LDH during the co-precipitation reaction (Fig. 1).

## Characterization of selectively encapsulated curcuminoids (SEC)-LDH

### PXRD analysis

**Fig. 2:**
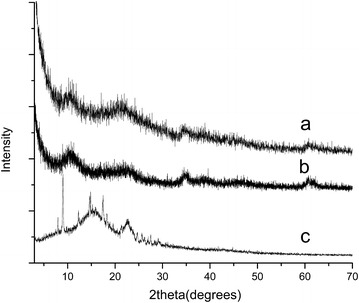
PXRD pattern for* a* SEC-LDH,* b* CIC-LDH and* c* curcuminoids. PXRD patterns were obtained for the dried powders of isolated curcuminoids, chemically isolated curcuminoids encapsulated LDH (CIC-LDH), and the LDH prepared by curcuminoid encapsulation using the in situ novel method

PXRD analysis was used to understand the successful and selective intercalation of curcuminoids from natural turmeric into the LDH, and the pattern (Fig. 2) was compared with that of CIC-LDH and isolated curcuminoids. The LDH resulted by the encapsulation of curcuminoids through different routes demonstrated similar structural characteristics such as the peak positions and the peak intensities of both basal and non basal reflections. It was observed that for both CIC-LDH and SEC-LDH, the basal reflection (003) appears at a 2 theta value of 11.5°, and the corresponding inter-planar spacing is confirmed as 0.76 nm. There is no appearance of peaks related to the presence of any crystalline curcuminoids.

The possible intercalation reaction of curcuminoids into LDH could be explained based on the structure of the curcumin, which is the main active component among curcuminoids. The structure of LDH consists of positively charged cation layers and anions in the interlayer spacing and water molecules. The keto-enol tautomerism of curcumin allows a negative charge to form on the curcumin structure at basic pH values; hence, as a result of that configuration, curcumin can be encapsulated into the inter-layer spacing during the co-precipitation reaction. Although curcumin molecules exhibit an overall hydrophobic nature, the presence of hydrophilic hydroxyl groups on the surface and the negative charges originated as a result of keto-enol tautomerism selectively driving the curcumin groups into the inter-layer spacing of LDHs. Similar behavior is followed by DMC and BDMC that are present in curcuminoids.

The width of curcumin is approximately 0.69 nm [13]. As for isolated pure curcuminoids intercalated LDH, during the selective encapsulation process, curcuminoids adapt to a flat molecule, where the plane of curcuminoids within the brucite layers arrange in a parallel orientation. Moreover, the intensity of the basal reflection is very low, while the peak is broad due to the disordering of the curcuminoids within the layers. Such disordering may occur due to the presence of different types of large organic molecules (turbostatic disordering) with a flexible ring structure. As a result, improved water solubility can be expected from the nanohybrid. Other researchers have also reported such improved solubility with synthetic curcumin-montmorillonite nanocomposites [19]. Further evidence for the interactions between the LDHs and curcuminoids are provided by the FTIR analysis (see Additional file 1).

### SEM and TEM analysis

**Fig. 3:**
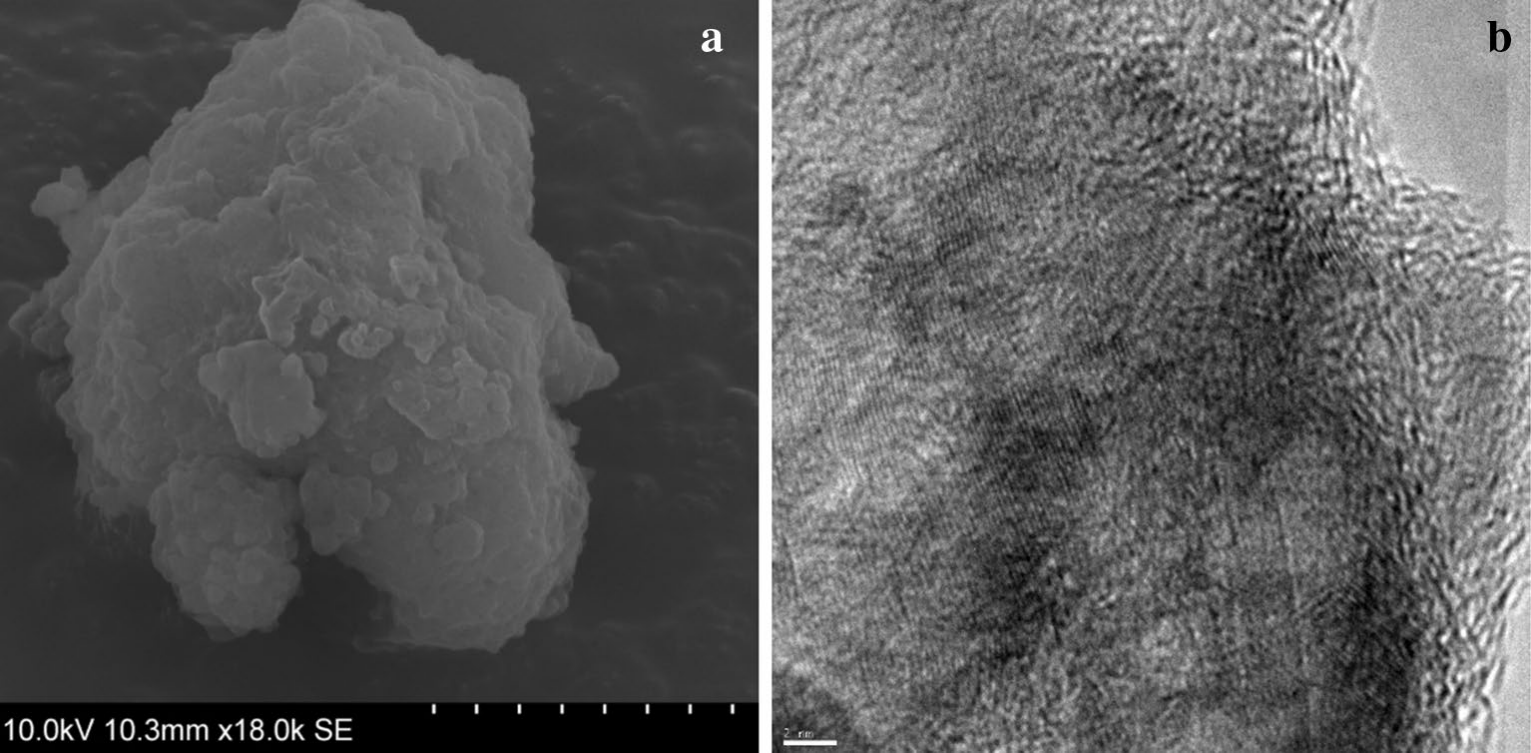
Electron microscopic images **a** SEM and **b** TEM of SEC-LDH. SEM image demonstrate the plate-like morphology and the TEM shows the internal structure, the* scale bar* represents 2 nm, the layered pattern is visible in **b**

As shown in Fig. 3a, the SEC-LDH demonstrates the typical plate-like morphology. The layered nature and lattice structure are clearly visible in Fig. 3b, and a basal spacing of 0.25 nm is suggested. This observation corroborates the basal spacing suggested by the PXRD analysis.

### Release behavior of SEC-LDH—effect of pH

**Fig. 4:**
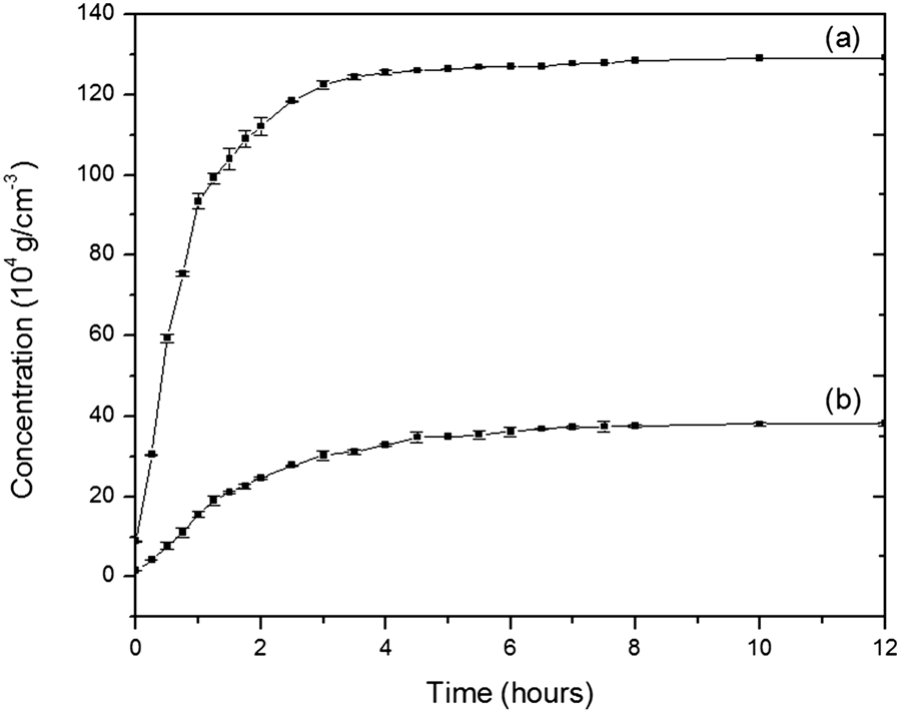
Release behavior of SEC-LDH at* a* pH 3,* b* pH 5 in aqueous medium. Release behavior of the nanohybrid was studied under acidic pH which is closer to the infected skin

The release properties of curcuminoids have been studied at pH 3 and 5. The release study was performed at these pH values because the pH of the intact skin is acidic. The release profiles for SEC-LDH at pH 3 and 5 are shown in Fig. 4. The release profile of SEC-LDH shows a high initial drug release rate in the first 3 h and then reaches an almost constant level over a longer period, which confirms the slow and sustained release of the drug. Such a release profile is characteristic of a diffusion-controlled release process [20].

Furthermore, the amount of curcuminoids released at pH 3 is significantly greater than that released at pH 5 because the pH 3 medium consists of more H^+^ ions than the pH 5 medium, which leads to a higher proton attack to the curcuminoid ions; thus, the curcuminoid ions become protonated, leading to a higher amount of curcuminoid ions released from the layered matrix to the medium. Meanwhile, no measurable release was observed for pure curcuminoids in an aqueous medium due to its very low solubility.

### Percentage intercalated and percentage release

It was found that the percentage of curcuminoids intercalated into the layered matrix was 72 %; however, only 43 % of the intercalated curcuminoids were released within the first 3 h. The concentration of curcuminoids released in 3 h was 0.0122 g cm^−3^ in pH 3 and 0.0030 g cm^−3^ in pH 5, and the concentration that remained after 10 h was 0.0128 g cm^−3^ in pH 3 and 0.0039 g cm^−3^ in pH 5. As a result, the SEC-LDH is expected to demonstrate long-term release in practical application.

### Release kinetics of SEC-LDH

The release mechanism of the curcuminoids from LDH was investigated referring to four different kinetic models, first order, zeroth order, Korsmeyer-Peppas and Higuichi. Rate constants (*k*) and r^2^ values were obtained from the best fit curves and are summarized in the supplementary materials.

The first order kinetic model resulted in, r^2^ values of 0.79 and 0.91 at pH values of 3 and 5, respectively suggesting that the release is not based on a dissolution mechanism. Rather the release behavior may happen according to several independent processes that occur based on the types of host guest attractions. These evidences suggest that there are various degrees of host guest interactions ranging from attractions between the intercalated curcuminoids and nanolayers to those between surface and layer edges with adsorbed curcuminoid molecules.

**Fig. 5:**
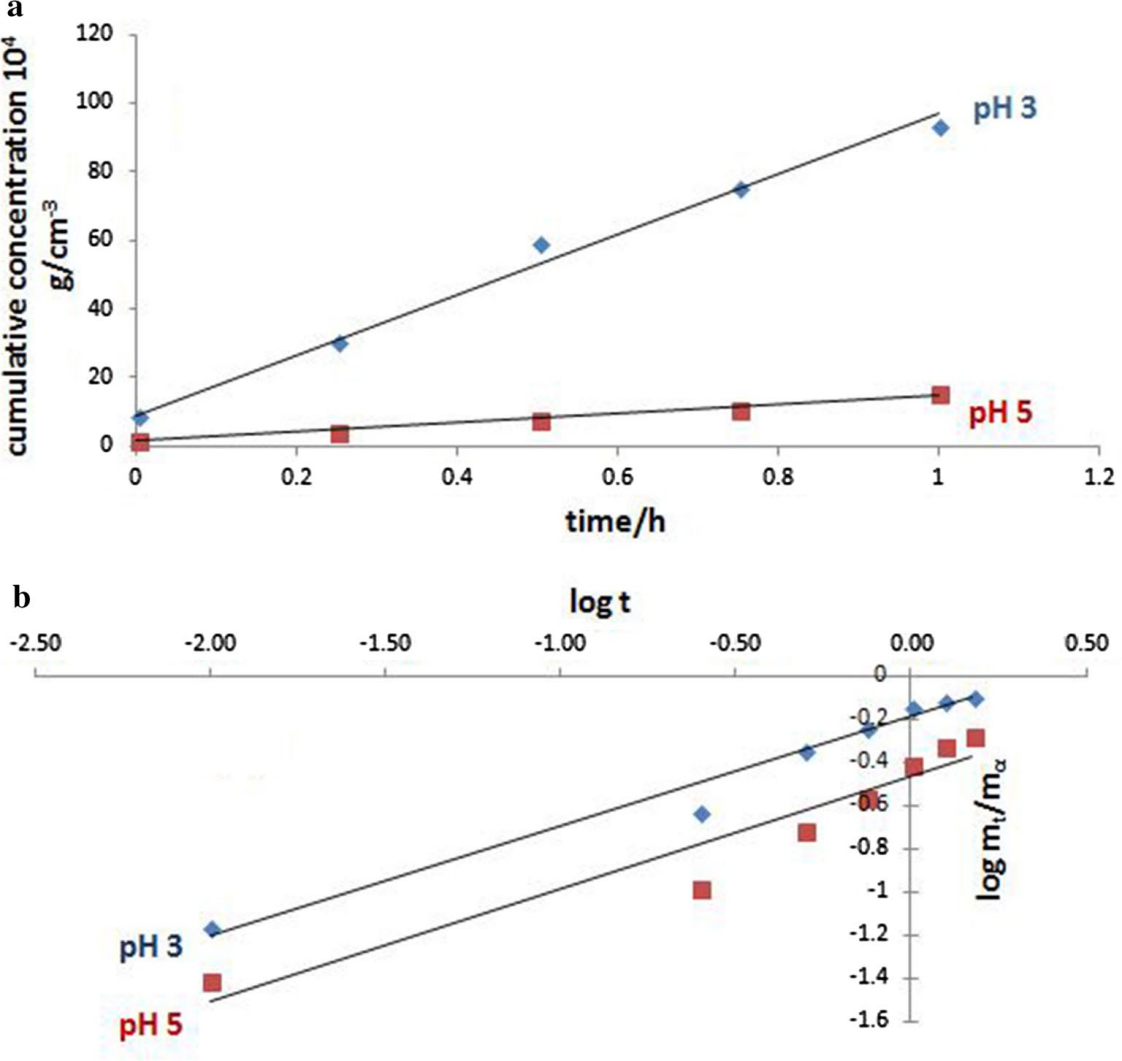
Kinetic behavior of the Sec-LDH according to the **a** zeroth order model, **b** Korsmeyer Peppas model

On the other hand, the zeroth order model and Korsmeyer-Peppas model provided *r*^2^ values of more than 0.90 at both pHs (see Fig. 5). These models have been accepted for many of the transdermal systems and matrix tablets which demonstrate a low solubility and coated drugs [12, 21–25]. According to these models, pharmaceutical dosage forms follow a release profile where the same amount of drug by unit of time is released; thus, it is the ideal method of drug release to achieve a pharmacological prolonged action. These observations therefore, agree with the prolonged release behavior of curcuminoids and their potential against number of microbes as observed in this study.

Conversely, Higuichi model describes drug release as a diffusion process based on Fick’s law,

$${\text{M}}_{\text{t}} /{\text{ M}}_{0} = {\text{ Kt}}^{\text{n}}$$

where M_t_ is the amount of material released at time t, M_0_ is the total amount of material added, k is the rate constant and n is the diffusion exponent related to the diffusion mechanism. According to Higuichi model, the *n* value is 0.5 for this system suggesting a Fickian diffusion release mechanism of curcuminoids.

Based on the results, a diffusion-controlled process or heterogeneous diffusion process is suggested for the curcuminoid-LDH system.

### Thermal stability

To study the thermal behavior of curcuminoids and SEC-LDH, analysis was conducted in a flowing nitrogen environment. In TGA analysis of SEC-LDH, three weight loss steps were observed, which contributed to a total weight loss of 48.56 %. The weight loss due to the removal of physisorbed and chemisorbed water was reported as approximately 23.32 % at a maximum temperature of 70 °C and extended up to ~125 °C. The amount of hydration was significantly low compared to other inorganic LDHs because SEC-LDH is less prone to being hydrated due to the intercalation of large organic anions. At the range of 200–350 °C, SEC-LDH showed a complete dehydroxylation of layers, together with partial combustion of the intercalated curcuminoids at the edges or surfaces of the crystallites, approximating to a weight loss of 16.18 %.

On the other hand, the decomposition peak does not appear to be sharp for SEC-LDH but is a broad peak in the range of 200–450 °C, thus indicating the different bonding environment of curcuminoids after the intercalation. However, it is difficult to distinguish between two weight losses—the dehydroxylation of nanolayers and curcuminoids decomposition within the LDH matrix. Meanwhile, for curcuminoids, a sharp decomposition peak is observed at 360 °C. This observation confirms that the intercalation of curcuminoids into the layered matrix increased the thermal stability of the curcuminoids. The LDH matrix thus improves the stability of the anions because it provides protection for the intercalated anions against thermal combustion.

### Photo-stability of SEC-LDH

**Fig. 6:**
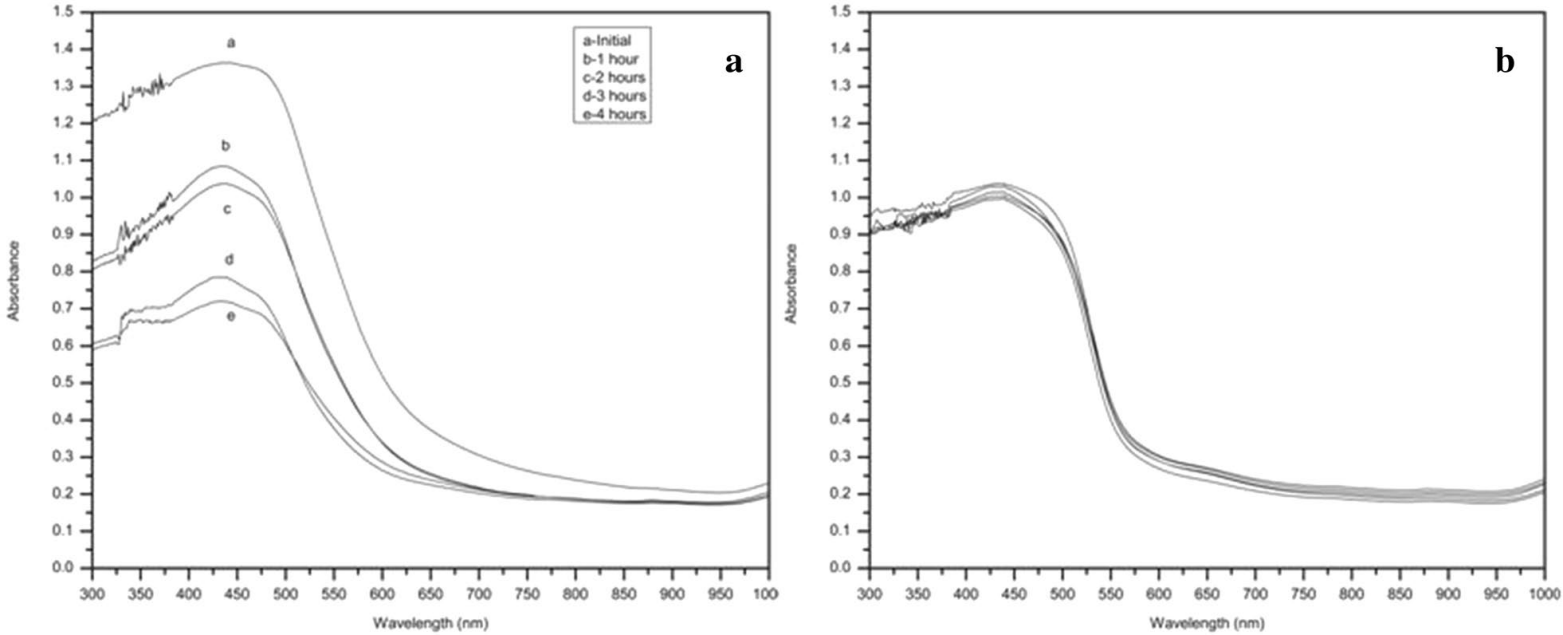
Solid state absorbance spectra of **a** curcuminoids **b** SEC-LDH. Absorbance measurements were carried out for the UV exposed sec-LDH at different time intervals

According to the observations, the photo-stability (Fig. 6) study of curcuminoids shows that the maximum absorbance wave length (λ_max_) has gradually shifted to a lower wave length, decreasing the absorbance at λ_max_ with the time of UV exposure. Compared with this, there is only a negligible decrease in the absorbance of SEC-LDH. Additionally, according to the A/A_0_ vs time graph, curcuminoids absorbance drops to a value that is nearly half of the initial. For SEC-LDH, this drop is also insignificant, confirming the protection of the molecule within a layered structure.

Additionally, λ_max_ is preserved over time. In SEC-LDH, curcuminoids exist in phenolate form; thus, electrostatic interactions and hydrogen bonds are formed with LDH layers. Furthermore, ketone and methoxy functional groups also form hydrogen bonds with hydroxide layers. These interactions result in the highly stabilized form of curcuminoids in between LDH layers. In addition to this, it has been found that photo-degradation of pure curcuminoids is enhanced due to the reaction of photo-excited curcuminoid molecules with molecular oxygen, which produces singlet oxygen [26]. These degradation reactions can be prevented due to various interactions within the LDH as explained below.

It has been found that the diketone moiety mainly accounts for the photo-degradation of these molecules. This process gives rise to different compounds, such as feruloyl methane, ferulic acid, vanillin and acetone. Initially, curcumin degrades into feruloyl methane and ferulic acid. Then, feruloyl methane further degrades into vanillin and acetone. Accumulating degradation products are also absorbed in the same wavelength range, but they are more photo stable. Therefore, curcuminoids degradation has a nonlinear rate.

### Antimicrobial properties

The antimicrobial activity of SEC-LDH was tested against *Staphylococcus aureus* (ATCC 25923)*, Escherichia coli* (ATCC 25922), and *Pseudomonas aeruginosa* (ATCC 27853), as well as two yeast species, i.e., *Candida albicans* (ATCC 10231) and *Candida dubliniensis* (Clinical isolate), in the presence of various pH (3, 4 and 5) conditions in triplicates. According to the observations, the extracted curcuminoids and SEC-LDH both showed inhibitory activity against the tested microbial species. When comparing the extracted curcuminoids and SEC-LDH (in both, the concentration of curcuminoids is 86 × 10^−3^ g cm^−3^), SEC-LDH showed a better inhibition zone for the tested organisms. No zone of inhibition was observed for the curcuminoid acetone extract.

According to the study findings, the average zone of inhibition given by SEC-LDH at pH 3, against three bacterial species and *C. albicans* was significantly greater than the average zone of inhibition of the control (p < 0.05). The mean zone exhibited by SEC-LDH at pH 4, against only for *P. aeruginosa* showed a statistically greater zone of inhibition than that of the control (p < 0.05). Further, the results suggest that SEC-LDH has an improved slow-release property against most of the tested microorganisms Table 1.

**Table 1:** Antimicrobial and antifungal studies

Substance tested	*Staphylococcus aureus* ATCC 25923 (mm)	*Pseudomonas aeruginosa* ATCC 27853 (mm)	*Escherichia coli* ATCC 25922 (mm)	*Candida albicans* ATCC 10231 (mm)	*Candida dubliniensis* clinical isolate (mm)
Positive control	11	11	23	11	21
Negative control	–	–	–	–	–
Pure turmeric	–	–	–	–	–
SEC-LDH (at pH 3)	17	19	27	21.3	8.3
SEC-LDH (at pH 4)	15.3	13	7.3	–	–
SEC-LDH (at pH 5)	8	10	–	–	–

Anti-microbial behavior of SEC-LDH composite was studied at pH 3, 4 and 5. Fluconazole was used as the positive control for the tested yeast species, whereas vancomycin and gentamicin were used as the positive controls for the tested bacterial species; and sterile acidic solvents and nitrate were used as negative controls

Fluconazole was used as the positive control for the tested yeast species, whereas vancomycin and gentamicin were used as the positive controls for the tested bacterial species. Sterile acidic solvents (pH 3, 4 and 5) were used as the negative controls.

The results show that at pH 4, SEC-LDH has a relatively large zone of inhibition for the *S. aureus* (ATCC 25923) and *P. aeruginosa* (ATCC 27853) species; furthermore, at pH 3, *E. coli* (ATCC 25922) and *C. albicans* (ATCC 10231) show large inhibition zones.

Due to the sustained release of curcuminoids from SEC-LDH composites, a large inhibition zone was shown for SEC-LDH. No inhibition zone was observed for the extracted curcuminoids acetone even with the same concentration of curcuminoids. And also no inhibition zone was observed for pure nitrate-LDH.

The human skin is believed to be acidic. [27] *S. aureus*, *Candida species* are commensals on skin. This skin flora is mainly the source of wound infections and the origin is endogenous. The results gained from antibacterial and antifungal susceptibility testing show the effectiveness of SEC-LDH at acidic environments against the tested microorganisms in the current study.

## Experimental

All inorganic materials were of analytical grade and used without further purification. Turmeric was purchased from an Ayurveda pharmacy. In all the experiments, distilled water was used.

### Synthesis of selectively encapsulated curcuminoids-layered double hydroxide (SEC-LDH)

SEC-LDH was synthesized by adding the Mg–Al–NO_3_ solution (300 cm^3^ of the Mg–Al–NO_3_ solution was prepared by dissolving 1 mol dm^−3^ Mg (NO_3_)_2_·6H_2_O and 1 mol dm^−3^ Al (NO_3_)_3_ in a 2:1 ratio) drop-wise to a concentrated turmeric in acetone (40 g/200 cm^3^) under vigorous stirring conditions at 60 °C. During the addition period, the pH of the solution was maintained at 9 by adding 1 mol dm^−3^ NaOH. The slurry was then stirred overnight in a closed container at 60 °C. Finally, it was filtered and washed thoroughly with distilled water to remove impurities and dried at 90 °C.

### Characterization

Curcuminoids in acetone were tested using thin layer chromatography to determine the presence of different curcuminoids. Thin layer chromatography was carried out using chloroform: methanol mobile phase with a composition of 95:5. After development, the plates were removed and dried. Spots were analyzed. The synthesized SEC-LDH (1.0 g) was stirred in acetone (5 cm^−3^) overnight to extract all intercalated curcuminoids. The same procedure used for the crude turmeric for TLC analysis was repeated.

Powder X-ray diffraction (PXRD) patterns were recorded to confirm the formation of SEC-LDH to identify the structural orientation and crystalline phases in the synthesized nanocomposites. PXRD experiment was carried out using the Brucker D8 focus X-ray powder diffractometer using Cu Kα radiation (λ = 1.540 Å) over a 2θ angle from 2° to 70° with a step size of 0.02°.

Fourier transform infra-red (FTIR) spectra were recorded to identify the functional groups in the synthesized materials. The Nicolet IS 10 instrument was used to inspect the powdered sample using diffuse reflection mode in the range from 600 to 4000 cm^−1^. The sample was mixed with potassium bromide in 1:100 ratios, and then the mixture was ground to a fine powder. Furthermore, a disc having an even surface was prepared by compressing the powdered sample.

Thermo gravimetric analysis (TGA) was used to study the thermal profile of the material as a function of temperature to understand the thermal stability of the synthesized materials. The SDTQ 600 thermo gravimetric analyzer was used in this study. The sample (10 mg) was heated at a rate of 10 °C per min in a nitrogen atmosphere over a temperature range of 30–1000 °C. The Q series 600 was used for this analysis.

A UV-2602 single beam scanning spectrophotometer was used for the curcuminoid release studies as a function of time at different pH values, and the PerkinElmer Lambda35 UV/Vis spectrophotometer was used for solid UV analysis.

Morphological studies were carried out using scanning electron microscope (SEM) and transmission electron microscopy (TEM). SEM characterization was done using the secondary electron mode of SU6600 microscope. The sample was placed on an aluminium stub and sputtered with a thin layer of gold. TEM analysis was carried out using a JEOL JEM 2100 microscope operating at 200 keV. The samples were dispersed in methanol using ultrasonication for 5 min. The suspended nanoparticles were loaded onto Lecay carbon-coated copper grids (300 mesh), and the sample containing the grids was dried for 24 h at room temperature prior to observation.

### Release behavior of Mg–Al-curcuminoids-LDH

The release behavior of encapsulated curcuminoids from SEC-LDH was profiled in pH 3 and 5 buffer solutions. SEC-LDH powder (2.00 g) was dispersed in the buffer solution. The amount of release of curcuminoids was determined at 15 min intervals, followed by 30 min intervals of monitoring the variation of absorbance in the UV–Vis absorption spectroscopy, with the peak at 427 nm (λ_max_ of curcuminoids). To investigate the kinetics for the release behavior of curcuminoids from LDH, the data were fitted to the following kinetic models: the zero order release model, first order release model, Higuchi release model, and Korsmeyer-Peppas release model.

### The zeroth order rate model

1 $${\text{Q}}_{0} - {\text{Q}}_{\text{t}} = {\text{ K}}_{0} {\text{t}}$$

where Q_t_ is the amount of drug dissolved in time t, Q_0_ is the initial amount of the drug, K_0_ is the zero order release constant, and T is the time in h [25].

The first-order rate model.

2 $${\text{Log C}}_{\text{t}} = {\text{ Log C}}_{0} + {\text{ Kt}}/ 2. 30 3$$

where *C*_0_ is the initial concentration of the drug, C_t_ is the concentration of the drug after time t, K is the first order rate constant, and t is the time in h [25].

The Higuchi model

3 $${\text{Log Q }} = 1/2{\text{ log t }} + {\text{ log K}}_{\text{H}}$$

where Q is the amount of drug released, K_H_ is the Higuchi dissociation constant, and T is the time in h [25].

The Korsmeyer-Peppas model

4 $${\text{M}}_{\text{t}} /{\text{M}}_{\infty } = {\text{ Kt}}^{\text{n}}$$

where M_t_/M_∞_—fraction of drug released at time t, n-diffusion exponent, K-kinetic constant, and the assumption is infinite time, i.e., 10 h.

### Curcuminoids release properties in water

The dissolution test was performed at a constant temperature (37 ± 0.5 °C) by suspending SEC-LDH nanocomposites (2.00 g) in a phosphate buffer (50 cm^−3^) at various pH values (3 and 5). Aliquots (2.00 cm^−3^) of supernatant were taken at 15 min intervals, followed by 30 min intervals, and the curcuminoids content was determined via UV absorption at λ = 427 nm.

### Percentage intercalated and percentage release of curcuminoids

Determination of the total amount of intercalated curcuminoids was carried out by dissolving the curcuminoids that were intercalated in the SEC-LDH composite (2 g) in acetone (15 cm^3^) and stirring overnight. After 24 h, the absorbance of the filtrate was measured by using a UV–Vis spectrometer, and the concentration of curcuminoids intercalated was determined to be λ_max_ of 427 nm.

$$\begin{aligned}&Percentage\, intercalated \\ &\quad= \frac{percentage\, of \,curcumin\, intercalated\, in\, SEC - LDH}{Total\, percentage\, of\, curcumin\, used}\\ &\quad\quad\times 100\end{aligned}$$

Determination of the amount of released curcuminoids was carried out by suspending SEC-LDH (2 g) in a phosphate buffer solution (pH 3) for 24 h. An aliquot (2.00 cm^3^) was taken, and the released amount of curcuminoids was determined via UV absorption at a wavelength of 427 nm.

$$\begin{aligned}&Percentage\, released \\ &\quad= \frac{percentage\, of\, curcumin\, released\, from\, SEC - LDH}{concentration\, of\, curcuminoid\, encapsulated} \\ &\quad\quad\times 100 \end{aligned}$$

### Photo-stability study of curcuminoids and SEC-LDH

Four SEC-LDH samples (1.0 g) were exposed to UV light (wavelength 365 nm) for 1, 2, 3 and 4 h; they were placed in a black box during the exposure. One equal weight sample was kept in the dark throughout the whole experiment. Each sample was scanned using the solid state attachment of a UV visible spectrophotometer. The samples were diluted with spectroscopic grade BaCl_2_ prior to scanning. The same procedure was repeated for the crude curcuminoid sample.

### Determination of antimicrobial activity

The antimicrobial activity of the SEC-LDH composite was tested against three bacterial species (*S. aureus* (ATCC 25923)*, E. coli* (ATCC 25922)*, P. aeruginosa* (ATCC 27853)) and two fungal species [*C. albicans* (ATCC 10231) and *C. dubliniensis* (clinical isolate)] using the agar well diffusion method [24]. For each tested bacterial and yeast strain, a suspension was prepared in sterile normal saline and was turbidly adjusted to the McFarland 0.5 turbidity standards.

One milliliter of the test inoculum was inoculated on a solidified MHA (Oxoid, England) plate to obtain a confluent growth. Using a sterile cork-borer, 9 mm wells were cut out of each MHA plate. The bottoms of the wells were sealed by adding a drop of molten agar into the wells using a sterile pipette.

The pH of the SEC-LDH composite was adjusted (pH 3, 4 and 5). Wells were loaded with 180 µl of the SEC-LDH composite by using a micropipette. Fluconazole was used as the positive control for the tested yeast species, whereas vancomycin and gentamicin were used as the positive controls for the tested bacterial species; and sterile acidic solvents (pH 3, 4 and 5) were used as negative controls. Plates were kept at room temperature for nearly 10 min and then incubated aerobically at 37 °C and observed after 24 h.

The antimicrobial activity of the SEC-LDH composite was tested in triplicates and the mean diameter of the zone inhibition zone was recorded. The statistical analysis was carried out by using the software, Statistical Package for Social Sciences (SPSS) version 20.0. One sample t test was used for quantitative variables. The level of significance was taken at 5 % (p < 0.05).

## Conclusions

PXRD and FTIR data revealed the successful selective encapsulation of natural curcuminoids into the nanolayers of the LDH. SEM and TEM images confirmed the typical hexagonal morphology and the layering pattern of the resulting nanohybrid. TGA and UV exposure data proposed the stabilization of the curcuminoid molecules within the nanolayers, thus making them suitable for potential practical application. Slow and sustained behavior of the encapsulated curcuminoids was observed in acidic pH values, thus proving their applicability in antibacterial skin formulations. The release data fit the zero order kinetic model, thus suggesting that the release mechanism is based on drug dissolution from dosage forms that do not disaggregate and release the drug in a slow and sustainable manner. Improved and sustained activity of the novel nanohybrid proved the antimicrobial activity against the 3 bacteria *species* and 2 candida *species*. In this regard, the SEC-LDH nanocomposites can provide a powerful route for developing a new efficient drug delivery system with a suspended release rate.

## Additional file

10.1186/s13065-016-0179-7 PXRD pattern of nitrate LDH (for comparison purposes). **Figure S2.** FTIR spectra for (A) curcuminoids (B) SEC-LDH. **Table S1.** Peak assignments vs peak positions bonds. **Figure S3.** Thermograms of the (a) curcuminoids and (b) SEC-LDH. **Table S2.** Parameters for kinetic models. **Figure S4.** Kinetic models for the behaviour of SEC-LDH at pH 3 and 5.
