# Supplementary material for: Natural curcuminoids encapsulated in layered double hydroxides: a novel antimicrobial nanohybrid
Source: Chem Cent J. 2016 May 31;10:35. doi: 10.1186/s13065-016-0179-7 (PMC4888422; doi:10.1186/s13065-016-0179-7)
Supplement: Supplementary file 1 — 10.1186/s13065-016-0179-7 PXRD pattern of nitrate LDH (for comparison purposes). Figure S2. FTIR spectra for (A) curcuminoids (B) SEC-LDH. Table S1. Peak assignments vs peak positions bonds. Figure S3. Thermograms of the (a) curcuminoids and (b) SEC-LDH. Table S2. Parameters for kinetic models. Figure S4. Kinetic models for the behaviour of SEC-LDH at pH 3 and 5. [file 13065_2016_179_MOESM1_ESM.docx]

**Additional File 1**

**Characterization of nanohybid**

**PXRD analysis of LDH only**

Nitrate anions have been used as the guest anions to synthesize the LDH.

**Figure S1. PXRD pattern of nitrate LDH (for comparison purposes)**

**FTIR analysis**

FTIR spectra of curcuminoids (Fig S1 (a)) demonstrate relatively sharp bands due to low levels of intra- and inter-molecular hydrogen bonding. The significant peaks of curcuminoids are the bands at 3370 cm^-1^-3380 cm^-1^ corresponding to the phenolic O-H stretching vibrations, the peak at 1700 cm^-1^ representing the stretching vibration of the benzene ring skeleton, the peak at 1510 cm^-1^ corresponding to mixed C=O, C=C vibrations, and the peak at 1280 cm^-1^ corresponding to the Ar-O stretching vibration. After the encapsulation within the LDH layers, these peaks shift as follows.

The peak for O-H stretching in the 3370 cm^-1^-3380 cm^-1^ range is significantly broadened after intercalation such that in LDH, that range extends from 3360 cm^-1^ to 3380 cm^-1^. This observation confirms that within the LDH nanolayers, O-H groups are further stabilized, indicating a strong hydrogen bonding network between curcuminoid O-H groups, hydroxyl groups of metal hydroxides and interlayer water molecules. In addition to O-H stretching, the C=O stretching and methoxy C-O stretching vibrations of curcuminoids within LDH also shift to low frequencies of 1600 cm^-1^ and 1270 cm^-1^ from 1700 cm^-1^ and 1280 cm^-1^, respectively. This indicates a decrease of the electron density of the bonds, compensating for the formation of hydrogen bonds between H bond donor oxygen atoms and LDH layers, as well as intermolecular water molecules. Furthermore, the weak peak for the mixed C=C and C=O stretching of curcuminoids at 1510 cm⁻¹ shifts to 1500 cm⁻¹ in LDH, simultaneously forming a prominent sharp peak owing to the polarized carbonyl C=O bond, which was previously involved in intramolecular hydrogen bonding with the enolic O-H group and in LDH, in hydrogen bonding with metal hydroxide O-H groups. In addition to the above changes, the peak for aromatic C-C stretching shifts from 1430 cm^-1^ to 1390 cm^-1^. Additionally, the peak is broadened, confirming that the electron cloud of the aromatic rings is also involved in interactions that lead to stabilization of curcuminoid molecules. In addition to the curcuminoids peaks, SEC-LDH also has an additional peak at 815 cm^-1^, which corresponds to metal-oxygen stretching, indicating the intercalation of curcuminoids in the Mg-Al double hydroxide layered structure. According to all the data, curcuminoid molecules are strongly stabilized by the changing electron density within the intercalated conditions of SEC-LDH.





**Figure S2. FTIR spectra for (A) curcuminoids (B) SEC-LDH**

**Table S1: Peak assignments vs peak positions bonds**

| **Peak assignment** | **Curcuminoids** | **SEC-LDH** |
| --- | --- | --- |
|  | **Peak position cm^-1^** | **Peak position cm^-1^** |
| Phenolic OH stretching vibrations  Carbonyl stretching  Mixed C=C and C=O stretching  Aromatic C-C stretching  C-O stretching of methoxy groups | 3370-3380  1700  1510 (Weak peak)  1430    1280 | 3360-3380  1600  1510 (Strong sharp peak)  1390  1270 |

**Thermogravimetric analysis**


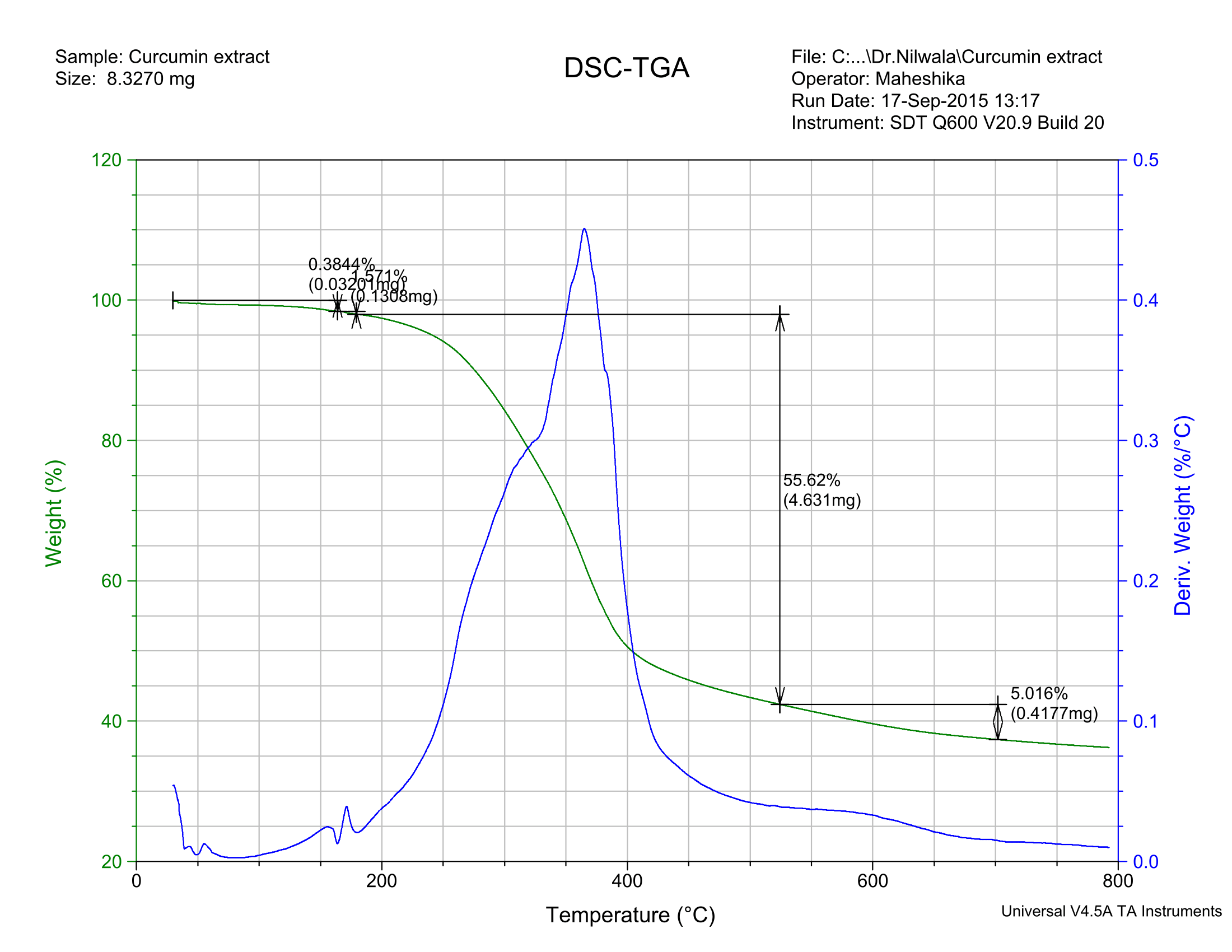


**(a)**


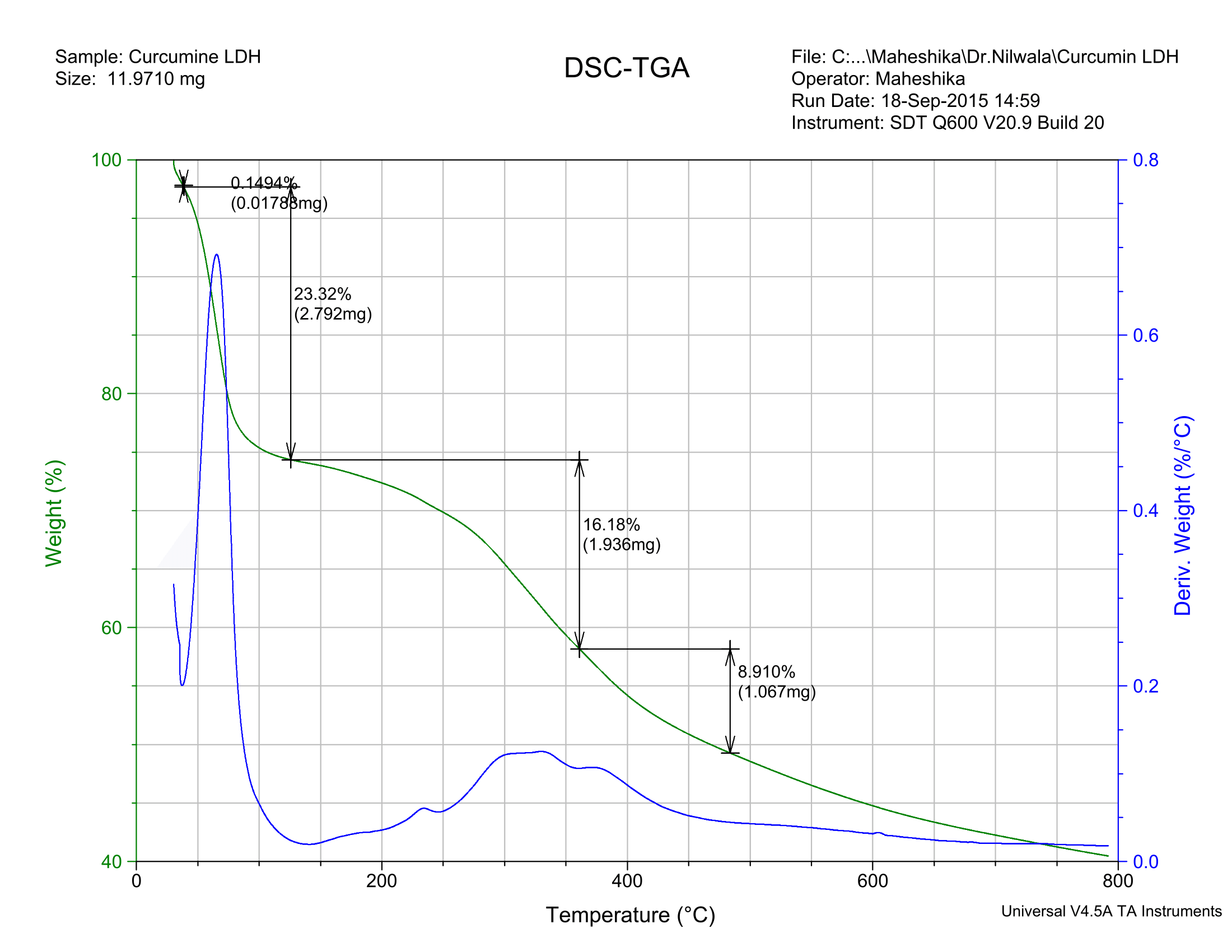


**(b)**

Fig S-2: TGA of (a) curcuminoids and (b) SEC-LDH

**Figure S3. Thermograms of the (a) curcuminoids and (b) SEC-LDH**

| Kinetic equations | pH 3 | pH 5 |
| --- | --- | --- |
| Zero order model Q_t_= K_0_t + Q_0_ | | |
| *k* (mol dm^-3^ s^-1^) | 88.037 | 13.213 |
| *r^2^* | 0.9884 | 0.9148 |
| First order model Log C_t_ = Log C_0_ +Kt/2.303 | | |
| *k* (s^-1^) | 2.782 | 2.572 |
| *r^2^* | 0.7974 | 0.9148 |
| Higuchi model Log Q = ½ log t + log K_H_ | | |
| *k* (mol dm^-3^ s^-1/2^) | 86.366 | 13.012 |
| *r^2^* | 0.9363 | 0.8612 |
| \| Korsmeyer Peppas model M_t_ /M _α_ = Kt_n_ \| \| --- \| | | |
| *k* (s^-1^) | 1.5392 | 2.9505 |
| *r^2^* | 0.9748 | 0.9179 |

**Release Kinetics of SEC-LDH**

**Table S2: Parameters for kinetic models**

**Release kinetic models of SEC-LDH**


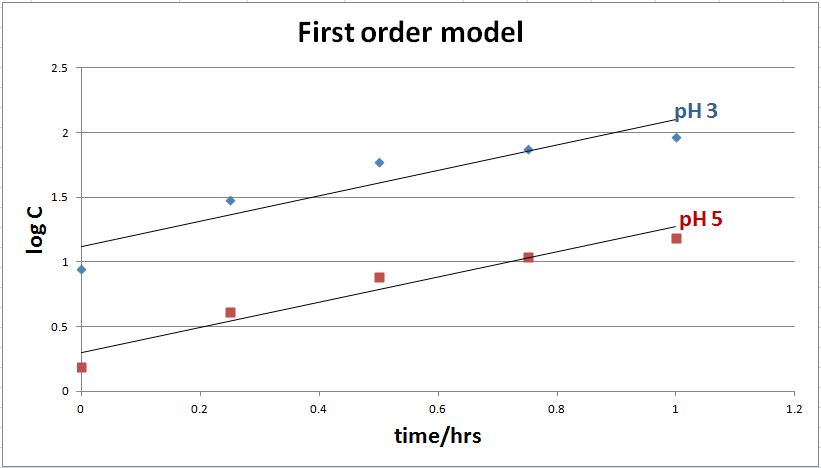

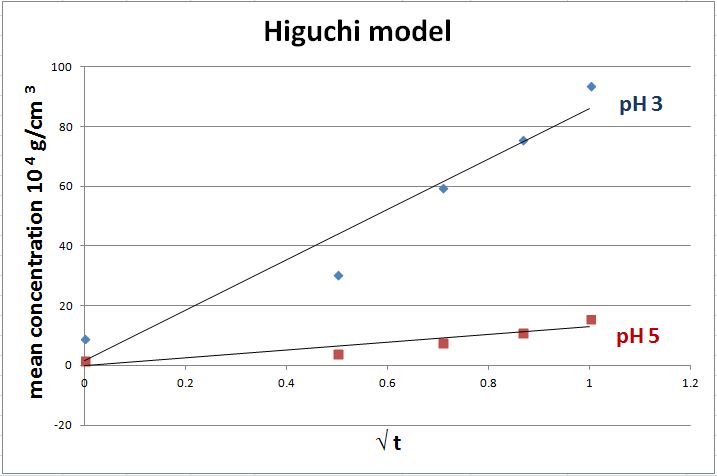


**Figure S4. Kinetic graphs for the release behavior of SEC- LDH at pH 3 & 5**

These models does not fit to the release behavior of curcuminoid from SEC-LDH.

The release pattern of the curcuminoid LDH does not follow these kinetic models.
